# Supplementary material for: Identification of Freezing-Responsive microRNAs and Their Targets in Chinese Jujube by Small RNA and Degradome Sequencing
Source: Int J Mol Sci. 2026 Sep 4;27(17):7912. doi: 10.3390/ijms27177912 (PMC13566890; doi:10.3390/ijms27177912)
Supplement: Supplementary file 1 [file ijms-27-07912-s001.zip › Supplementary Material S6.pdf]

Table S6. The detailed information of DEG miRNAs between LCK and LT

ID	Pvalue	FDR	log2FC	regulated
bcy-miR156	0.004513856	0.026277088	-1.360619107	down
bgy-miR156	0.004513856	0.026277088	-1.360619107	down
cpa-miR156e	0.00292721	0.020059756	-1.414614011	down
csi-miR159c-3p	0.000377791	0.005017565	1.577954784	up
ctr-miR319	0.000397154	0.005017565	1.576870111	up
fve-miR156g-5p	0.011502582	0.042133053	-1.162966269	down
gma-miR319a	5.00E-05	0.001145771	1.849872153	up
gma-miR319m	4.40E-05	0.001145771	1.925665006	up
gma-miR5368	0.006027541	0.03119013	-4.381221567	down
gra-miR157a	0.011502582	0.042133053	-1.162966269	down
gra-miR157b	0.011502582	0.042133053	-1.162966269	down
hbr-miR156	0.011502582	0.042133053	-1.162966269	down
hbr-miR159a	0.000116726	0.001729672	1.60345074	up
hbr-miR319	5.79E-05	0.001145771	1.940663328	up
lus-miR156h	0.00292721	0.020059756	-1.414614011	down
mes-miR156h	0.00292721	0.020059756	-1.414614011	down
mes-miR156i	0.00292721	0.020059756	-1.414614011	down
mes-miR156j	0.00292721	0.020059756	-1.414614011	down
mtr-miR319b-3p	5.00E-05	0.001145771	1.849872153	up
novel_miR_100	0.016566619	0.048715585	-7.940147546	down
novel_miR_104	0.010184764	0.042133053	-2.304416888	down
novel_miR_107	0.001214849	0.01131545	-1.366379254	down
novel_miR_110	0.017352083	0.048993395	-7.909123616	down
novel_miR_111	0.015299469	0.047260074	-1.846844372	down
novel_miR_112	0.014804271	0.047260074	-8.02423798	down
novel_miR_113	0.000535127	0.006461158	-9.886268498	down
novel_miR_122	0.003199716	0.020059756	1.229855439	up
novel_miR_130	0.001135218	0.010884734	-5.178767782	down
novel_miR_134	0.001554778	0.013233533	-5.620355545	down
novel_miR_139	2.25E-05	0.001145771	1.106216207	up
novel_miR_143	0.013693376	0.045233647	-8.093969955	down
novel_miR_144	0.010911998	0.042133053	-8.216635012	down
novel_miR_147	0.016587208	0.048715585	-2.566130442	down
novel_miR_150	0.005653058	0.029880262	-5.134878244	down
novel_miR_155	0.002023476	0.016491333	-5.915044252	down
novel_miR_157	0.006462484	0.032411841	-7.274088188	down
novel_miR_163	0.005682749	0.029880262	-3.111115727	down
novel_miR_164	0.015241323	0.047260074	-7.998155921	down
novel_miR_165	0.001519221	0.013233533	1.448722484	up
novel_miR_168	0.013545343	0.045233647	1.059049192	up
novel_miR_176	0.000997021	0.010386358	-1.129973137	down

novel_miR_177	0.00110659	0.010884734	-8.121646836	down
novel_miR_180	0.012313132	0.04318644	-8.142365998	down
novel_miR_183	0.015511742	0.047260074	-8.009771446	down
novel_miR_184	0.00101952	0.010386358	-7.255316733	down
novel_miR_187	0.000400174	0.005017565	-2.194495584	down
novel_miR_197	0.012952594	0.0449207	-8.150058973	down
novel_miR_198	0.003199716	0.020059756	1.229855439	up
novel_miR_204	0.009538042	0.042133053	-6.023638539	down
novel_miR_207	0.012320058	0.04318644	-8.137565469	down
novel_miR_210	0.007269169	0.03536939	-7.20645504	down
novel_miR_212	0.005423158	0.029465827	-5.318298773	down
novel_miR_213	7.80E-15	2.54E-12	-4.035809572	down
novel_miR_215	0.015400011	0.047260074	-8.008713547	down
novel_miR_221	0.011354021	0.042133053	-8.192918147	down
novel_miR_222	0.01577883	0.04762869	-7.973750133	down
novel_miR_225	0.008932316	0.041013171	-8.365350901	down
novel_miR_229	0.000615382	0.007164803	-4.390124083	down
novel_miR_231	0.002978803	0.020059756	-6.194065995	down
novel_miR_27	0.003199716	0.020059756	1.229855439	up
novel_miR_29	0.006405816	0.032411841	-8.548322535	down
novel_miR_3	0.004050094	0.02445057	-3.108749491	down
novel_miR_30	0.002978803	0.020059756	-6.194065995	down
novel_miR_31	0.013448257	0.045233647	-8.090976913	down
novel_miR_33	0.014635727	0.047260074	-8.026305849	down
novel_miR_35	0.005343996	0.029465827	-8.667059951	down
novel_miR_36	0.005131757	0.029350047	-8.682189168	down
novel_miR_4	0.013736598	0.045233647	-3.439558443	down
novel_miR_40	0.008595222	0.040029178	-7.0905325	down
novel_miR_41	0.011804973	0.042598879	-8.16692004	down
novel_miR_43	0.007956453	0.038144171	-6.140050649	down
novel_miR_48	0.000803224	0.009029344	-5.086876113	down
novel_miR_53	0.010184764	0.042133053	-2.304416888	down
novel_miR_55	0.017973273	0.048993395	-7.903517622	down
novel_miR_64	0.000920131	0.009998755	-1.750053298	down
novel_miR_65	0.011891098	0.042598879	-8.162205865	down
novel_miR_69	0.01649258	0.048715585	-7.9423454	down
novel_miR_7	0.005423158	0.029465827	-5.318298773	down
novel_miR_70	0.015184904	0.047260074	-7.999210702	down
novel_miR_73	2.36E-05	0.001145771	-3.885972141	down
novel_miR_75	0.008139276	0.038455128	-7.121623313	down
novel_miR_79	6.77E-13	1.10E-10	1.194822449	up
novel_miR_80	0.003516752	0.021631341	-8.897937701	down
novel_miR_87	0.018034378	0.048993395	-7.880857629	down
novel_miR_88	0.0031416	0.020059756	1.230541665	up

novel_miR_95	0.000282351	0.004002017	-7.90739674	down
novel_miR_96	0.014917644	0.047260074	-3.338020696	down
nta-miR319b	4.50E-05	0.001145771	1.930690557	up
ppe-miR156g	0.00292721	0.020059756	-1.414614011	down
ptc-miR156g	0.011502582	0.042133053	-1.162966269	down
ptc-miR156i	0.011502582	0.042133053	-1.162966269	down
ptc-miR156j	0.011502582	0.042133053	-1.162966269	down
ptc-miR319a	6.33E-05	0.001145771	1.950889063	up
ptc-miR319c	5.50E-05	0.001145771	1.943712708	up
rco-miR156h	0.011502582	0.042133053	-1.162966269	down
rco-miR319a	5.79E-05	0.001145771	1.940663328	up
tcc-miR156e	0.011502582	0.042133053	-1.162966269	down
vun-miR319a	5.00E-05	0.001145771	1.849872153	up
vvi-miR156g	0.011502582	0.042133053	-1.162966269	down
vvi-miR156i	0.011502582	0.042133053	-1.162966269	down
vvi-miR319c	9.81E-05	0.00168311	1.994071088	up
vvi-miR319f	0.000107627	0.001729672	1.992495481	up
